# Frequency of CFTR variants in southern Brazil and indication for modulators therapy in patients with cystic fibrosis

**DOI:** 10.1590/1678-4685-GMB-2020-0275

**Published:** 2021-12-06

**Authors:** Eliandra da Silveira Lima, Luíse Sgarabotto Pezzin, Ana Carolina Fensterseifer, Leonardo Araújo Pinto

**Affiliations:** ¹ Pontifícia Universidade Católica do Rio Grande do Sul, Pós-graduação em Pediatria e Saúde da Criança, Porto Alegre, RS, Brazil.; 2 Pontifícia Universidade Católica do Rio Grande do Sul, Escola de Medicina, Porto Alegre, RS, Brazil.

**Keywords:** Cystic fibrosis, CFTR, causing variant, personalized medicine

## Abstract

This is a descriptive cross-sectional study that aims to determine the distribution of the CFTR causing variant in a group of patients at a cystic fibrosis (CF) center in southern Brazil, as well as to describe causing variants that are treatable with mutation-specific drugs. Ninety-two patients from a CF reference center were assessed in this research, all of them with a clinical diagnosis of CF and both alleles identified with pathogenic variants. The most prevalent causing variants were F508del, R1162X, G542X, and N1303K. As for patients with a mutation-specific drug indication, 69.6 % were candidates for the use of Elexacaftor/Tezacaftor/Ivacaftor (Trikafta^®^), 44.6 % for the use of Tezacaftor/Ivacaftor (Symdeko ^®^), and 35.9 % for the use of Lumacaftor/Ivacaftor (Orkambi^®^). For the use of Ivacaftor (Kalydeco^®^), only two patients (2.2 %) were candidates following the Brazilian agency approval. According to the FDA, 10 patients would be candidates for Ivacaftor (10.9 %). Causing variants of classes I and II, which are related to a major severity of the illness, were identified in 135 of 184 alleles (73.3 %). In this study, more than 2/3 of the patients were candidates for the use of *CFTR* modulators therapy.

Cystic fibrosis (CF) is an autosomal recessive genetic disease, which is characterized by causing variants in the cystic fibrosis transmembrane conductance regulator (*CFTR*) gene, that is located in chromosome 7. CFTR is responsible for ions transport through the membrane of the epithelial cells (Ratjen et al., 2015; Athanazio et al., 2017).

The screening of the disease is done through the quantification of immunoreactive trypsinogen in the neonatal heel prick test. In the case of two positive dosages, the second one being done up to 30 days, an investigation with chloride dosage should be continued by quantitative methods in sweat (Athanazio *et al*., 2017).

The diagnosis of CF should also be considered when there is a compatible clinical picture, as meconium ileus, diarrhea, growth deficit, recurrent respiratory infections, nasal polyps, rectal prolapse, male infertility, electrolyte depletion, and positive family history.

For diagnostic confirmation, the individual must show alteration in two tests of chloride concentration in sweat, the value of which must be above 60 mmol/l. Values between 30 and 59 mmol/l are considered borderline. Another way to carry out confirmation is through the search for pathogenic variants, through panels, or sequencing of the *CFTR* gene, with the identification of mutations in two alleles (Athanazio *et al*., 2017).

Despite being a monogenic disease, it presents a great phenotypic variability (Ratjen et al., 2015). Currently, the variants of *CFTR* are categorized into six classes of causing variants, according to their functional effects: Class I results in no protein production; class II causes the retention of an unfolded protein in the endoplasmic reticulum, which results in the absence or diminished protein function; class III affects the regulation of the channel; class IV affects conductance; class V causes a significant reduction of mRNA or protein; class VI causes protein instability in the plasma membrane. Basically, all causing variants must disrupt the amount or functionality of the CFTR protein to cause CF. (Ratjen *et al.*, 2015; O’Sullivan and Freedman, 2009). This classification system has the advantage of characterizing causing variants by functional defect and helps to categorize the targets of new classes of molecular therapies (Brennan and Schrijver, 2015). 

The Brazilian population has some distinct characteristics that may be different from the European, due to its ethnic mixed features (Raskin *et al*., 2008). With the development of new therapies, which have specific genetic causing variants as targets, the knowledge of the local CFTR variation frequency is essential to improve patient care and for treatment cost estimative.

There are different classes of new therapies of CFTR modulators. The potentiator that acts by improving the CFTR function (for some variations of classes III to V); and the corrector that acts on the protein that is not expressed in the cell membrane (for classes I and II) (Athanazio *et al*., 2017; Brennan and Schrijver, 2015; Egan, 2016). In Brazil, ANVISA - the Brazilian Health Regulatory Agency - has approved the potentiator Ivacaftor (Kalydeco^®^), the combination of corrector/potentiator Lumacaftor/Ivacaftor (Orkambi^®^) and Tezacaftor/Ivacaftor (Symdeko ^®^). And, there is also the new Elexacaftor/Tezacaftor/Ivacaftor (Trikafta^®^), which has recently been approved in the United States (USA) by the Food and Drug Administration (FDA). 

At present, Ivacaftor is indicated for patients that have one of the 38 CFTR causing variants responsive to the *in vitro* test or to the clinical test. Under ANVISA’s approval, in Brazil, it is indicated for patients with one of the 9 class III causing variants, or patients older than 18 years with an R117H causing variant in the *CFTR*. Based on clinical studies only, Ivacaftor has proved to be effective especially for patients with G551D causing variant, providing a significant increase of FEV1, CFTR activity, and the decrease of the pulmonary exacerbation risk (Ramsey et al., 2011; Davies et al., 2013). In addition, the treatment with Ivacaftor has shown to be safe, with significant and sustained results in increasing FEV1 and the average distance in the walking test, and also in reducing the treatment with intravenous antibiotics and chloride in sweat (Salvatore et al., 2020). 

Lumacaftor, CFTR corrector, when associated with Ivacaftor, is indicated for patients with two years old or more that have both alleles with F508del causing variant. The combination Tezacaftor/Ivacaftor has recently been approved for use in Brazil and it is recommended for patients who are homozygous for F508del or for those who present at least one of the 26 causing variants that are responsive to the drug. Its use has shown significant effects, as the increase of the FEV1 percentage and life quality improvement (Boyle et al., 2014; Rowe et al., 2017). The new association of Elexacaftor/Tezacaftor/Ivacaftor has brought an increase in FEV1, reduction of pulmonary exacerbations and sweat chloride (Middleton et al., 2019).

The main objective of the present study is to determine the frequency of the CFTR causing variants in a group of follow-up patients from a multidisciplinary center of treatment for cystic fibrosis in southern Brazil, henceforth CF center (CFC). Likewise, it aims to describe the eligible causing variants for the use of specific causing variant therapies. The research also intends to compare the frequency of the most common causing variants found in the group with the ones described in the Brazilian registry of CF (GBEFC). 

This is a descriptive cross-sectional study, which includes CF-diagnosed patients with both mutated alleles from a reference center at Pontifícia Universidade Católica do Rio Grande do Sul (PUCRS). The variant identification was conducted in all patients with a clinical diagnosis or in those who presented changes in the neonatal screening test, with changes in the sweat test in two samples (chloride in sweat >60 mmol/l). The variant identification was conducted in all patients with a clinical diagnosis or in those who presented changes in the neonatal screening test, with changes in the sweat test in two samples (chloride in sweat >60 mmol/l). Patients with two borderline sweat tests (chloride between 30 - 59 mmol/l) and those symptomatic also continued to be investigated to identify CFTR variants.

Initially, patients were submitted to the isolated research of the F508del variant, which was available to all patients in the hospital. Heterozygous patients or those who did not have this variant identified kept being investigated through a causing variant panel with commercial kits. According to the availability of the healthcare plan, the carried-out panels investigated 11, 32, or 97 causing variants through reverse hybridization 

In patients from the public health system, the panel was performed using the Single-Nucleotide Primer Extension (SNaPshot) with a search for 11 causing variants. Genetic material was extracted from blood using the salting-out technique. The use of a standard marker (size calibrator - GeneScan120LIZ) ensures reproducibility. 

In cases in which the variation had not yet been identified in both alleles, genetic sequencing of all CFTR coding exons (Next-Generation Sequencing - NGS) was performed in a private laboratory (Mendelics Análise Genômica S.A., São Paulo, SP, Brasil), through a partnership between the GBEFC and the company Vertex. The collection of the oral swab sample was performed through a customized panel (Nextera Rapid Capture Mendelics Custom Panel V2) followed by next-generation sequencing with Illumina HiSeq. Alignment and identification of variants using bioinformatics protocols were performed with reference GRCh37 version of the human genome. Genetic sequencing by the NGS method simultaneously assesses the variation in both genes, being effective for the diagnosis of genetic diseases.

The molecular analyses used were validated and standardized as widely used for CFTR gene investigation. According to Athanazio *et al*. (2017), the initial analysis of the F508del variant is indicated as being the most common, followed by panels aimed at researching the most prevalent causing variants. In the case of non-identification in the previous tests, the CFTR sequencing must be used in the search for less frequent variants. 

The clinical and laboratory data were collected through physical or electronic records between February and October of 2019. The analysis considered the last set of information referring to nutritional status, FEV1, and laboratory tests from each patient.

Pancreatic insufficiency was considered through clinical symptoms, such as chronic diarrhea, steatorrhea, malnutrition, and through the dosage of fecal elastase by ELISA (<200 mcg/g). Following the guidelines of the European Society for Clinical Nutrition and Metabolism, weight and height, markers of pancreatic insufficiency, were measured in consultations every 3 months. The percentile of the body mass index for the age and weight-for-age were calculated through the programs WHO Anthro Survey Analyser and WHO AnthroPlus software by the age of the patient. 

The data were analyzed with the statistical software Statistical Package for the Social Sciences, version 18.0 for Windows (SPSS Inc., Chicago, IL, USA). The categorical variables were described as absolute frequency (number of cases) and relative frequency (% of every case). In addition, the continuous variables were described as mean and standard deviation or median and interquartile interval, following the data distribution. For the quantitative data with normal distribution, the Student's *t*-test was used for independent variables and the Mann-Whitney U test for the data with no normal distribution. The qualitative data were analyzed by the chi-square test, and, if necessary, Yates’s correction or Fischer’s exact test were used. 

The comparison between the prevalence of alleles for the most frequent causing variants at PUCRS and the GBEFC was performed with the chi-square test. All statistical tests used were two-tailed, and the significance level of 5% was established. This study was approved by the Ethics committee for Research of PUCRS and it is registered by the number 49692115.7.0000.5336.

This study assessed 92 patients of a single CFC (HSL/PUCRS). The median for patient age was 9.1 years old, 70 being children and adolescents (76.1 %). There was a slight predominance of the male gender (n=51; 55.4 %). The mean of FEV1 % pre-bronchodilator was 80.2 ± 30.5. Besides that, 56 % of patients presented bronchiectasis (n=35) and 90 % pancreatic insufficiency (n=82). The general characterization of the sample can be seen in Table 1.

**Table 1 -:** General characteristics of patients with Cystic Fibrosis in follow-up at the reference center for CF of São Lucas Hospital (PUCRS).

Variables	N= 92
Age (years), median (II)	9.1 (4.5 - 19.4)
Children and adolescents, N (%)	70 (76.1)
Gender, N (%)	
Male	51 (55.4)
Ethnicity, N (%)	
Caucasian	87 (94.6)
Age of diagnosis (years), median (II)	0.35 (0.1 - 3.5)
Nutritional status (adults)	
BMI (kg/m²), mean ± SD	21.4 ± 4.0
Nutritional Status (children and adolescents)	
Percentile Weight / Age, median (II)	35.8 (24.3 - 51.3)
Percentile BMI/ Age, median (II)	65.6 (39.6 - 84.3)
Causing variants classification, N (%)	
III-VI	22 (23.9)
I-II	70 (76.1)
Causing variants, N (%)	
F508del homozygous	33 (35.9)
F508del heterozygous	31 (33.7)
Others	28 (30.4)
Pancreatic Insufficiency, N (%)	82 (90.1)
Bronchiectasis, N (%)	35 (56.5)
Pulmonary Function, mean ± SD	
Predicted FEV_1_ %, pre-bronchodilator	80.2 ± 30.5

Table 2 contains the data on the prevalence of the main causing variants found in the CFC. The majority of the patients (n=70; 76.1 %) presented class I and II causing variants. The most prevalent causing variants of the CFC were F508del, R1162X, G542X, and N1303K. When compared to the GBEFC, it was observed that the highest prevalence of F508del, R1162X, and N1303K, in patients seen at the outpatient clinic with a statistically significant difference for all of them.

**Table 2 -:** Comparison between the prevalence of alleles for the most frequent causing variants in the CF Reference Center at São Lucas Hospital - PUCRS and Brazilian Registry of CF.

Causing variant (HGVS nomenclature)	Reference Center	GBEFC	P Value
	N (%)	N (%)	
F508del (NP_000483.3:p.Phe508del)	97 (52.7)	3578 (43.9)	<0.05
R1162X (NP_000483.3:p.Arg1162Ter)	14 (7.6)	163 (2.0)	<0.01
G542X (NP_000483.3:p.Gly542Ter)	9 (4.9)	541 (6.6)	NS
N1303K (NP_003117.2:p.Asn1303Lys)	7 (3.8)	101 (1.2)	<0.01
2184delA (NP_000483.3:p.Lys684fs)	3 (1.6)	58 (0.7)	NS
2184insA (NP_000483.3:p.Gln685fs)	2 (1.1)	19 (0.2)	NS
2789+5G>A (NM_000492.4:c.2657+5G>A)	2 (1.1)	28 (0.3)	NS
3120+1G>A (NM_000492.3:c.2988+1G>A)	2 (1.1)	224 (2.8)	NS
3132delTG (NP_000483.3:p.Val1001fs)	2 (1.1)	8 (0.1)	<0.01
3171delC (NP_000483.3:p.Tyr1014fs)	2 (1.1)	2 (0.0)	<0.01
3272-26A>G (NM_000492.4:c.3140-26A>G)	2 (1.1)	71 (0.9)	NS
711+5G>A (NM_000492.4:c.579+5G>A)	2 (1.1)	22 (0.3)	NS
Del Exons 19 - 21	2 (1.1)	12 (0.1)	<0.05
R347H (NP_004422.2:p.Arg347His)	2 (1.1)	11 (0.1)	<0.05

CF = cystic fibrosis; GBEFC = Brazilian study group of cystic fibrosis; N = number of alleles; % = percentage of alleles; P value calculated by the chi-square test with Yates correction; NS = not significant.

The most common characteristics of the *CFTR* causing variant, classes of causing variant, gender, and age of the CFC participants are described in Table 3. Found in 64 patients (69.6 %), 33 being homozygous (35.9 %), the most prevalent causing variant was F508del, which is considered from class II. The five main combinations of alleles found in this group were composed of one F508del allele and a class I or II allele. Pancreatic insufficiency was observed in most of these patients. In terms of the nutritional evaluation, the percentile of body mass index (BMI) by age revealed that all of them were eutrophic. In terms of lung function, a variation of FEV1 % was observed.

**Table 3 -:** Clinical, nutritional, and pulmonary function characteristics according to genotyping (including only patients with at least one F508del allele).

N	Allele 1	Class	Allele 2	Class	IP	pBMI/A	FEV1
33	F508del	II	F508del	II	32	62.7 ± 25.0	84.0 ± 37.0
6	F508del	II	R1162X	I	6	53.7 ± 33.2	69.0 ± 32.7
6	F508del	II	G542X	I	6	43.0 ± 25.8	101.0 ± 39.8
4	F508del	II	N1303K	II	4	64.8 ± 12.8	106.3 ± 4.6
2	F508del	II	2184insA	I	2	48.0	32.0
2	F508del	II	3272-26A>G	V	2	62.9 ± 9.3	63.0

N = number of genotypes; IP = pancreatic insufficiency; pBMI/A = percentile of body mass index by age; FEV_1_ = forced expiratory volume in the first second.

Concerning the patients with the indication for the use of specific-mutation drugs (Table 4), 69.6 % were candidates for the use of Elexacaftor/Tezacaftor/Ivacaftor (Trikafta^®^), 44.6 % for the use of Tezacaftor/Ivacaftor (Symdeko ^®^), and 35.9 % for the use of Lumacaftor/Ivacaftor (Orkambi^®^). Only two (2.2 %) were considered candidates for the use of Ivacaftor (Kalydeco^®^), according to the Brazilian approval for the use of the drug. 

**Table 4 -:** Candidate patients for the use of specific-causing variant drugs.

Drug	Candidate patients ANVISA (%)	Indicated causing variants ANVISA (n)
Elexacaftor/Tezacaftor/Ivacaftor (Trikafta^®^)	64 (69.6%)^*^	F508del homozygous (33)^*^ F508del heterozygous (31)^*^
Tezacaftor/Ivacaftor (Symdeko^®^)	41 (44.6%)^**^	F508del homozygous (33) 2789+5G>A (2), D1152H (1), L206W (1) , R347H (2), 3272-26A>G (2)^**^
Lumacaftor/Ivacaftor (Orkambi^®^)	33 (35.9%)	F508del homozygous (33)
Ivacaftor (Kalydeco^®^)	2 (2.2%)	R117H (1) e G551D (1)

^*^ Drug not yet approved by ANVISA. FDA-based indications.

^**^ Drug recently approved by ANVISA, on January 27th of 2020, with no information about indicated causing variant.

At the center, three patients are currently using modulating treatments (1 Ivacaftor, 1 Lumacaftor/Ivacaftor, and 1 Tezacaftor/Ivacaftor). In addition to the age indications and presence of the variants expressed for each drug, there are other recommendations for the patient to be a candidate for this use according to GBEFC. The patient should be linked to a specialized care center, he/she must have good adherence to the treatment and manifestations of respiratory disease or nutritional impairment, such as functional loss of FEV1 for 3 consecutive years, signs of bronchiectasis on chest tomography, frequent pulmonary exacerbations with the need for admission to hospital (>2 times/year), chronic sinus disease with clinical repercussion, malnutrition or BMI percentage <15 and chronic respiratory infection by typical CF bacteria. 

The most frequent causing variant found in this study was F508del, present in 97 identified alleles (52.7 %). However, the frequency was different than described in Northern Europe and the US, where F508del frequency may be as high as 87 % (Mateu et al., 2002). In the Tuscany region of Italy, a study found that 45 % of patients had at least one F508del allele identified, being more similar to the data found in this study (Terlizzi et al., 2019). The prevalence of homozygosis and heterozygosis of this causing variant reinforces the need for an initial screening for the F508del in patients with a suggestive clinical history or a confirmed diagnosis. On the other hand, the difference in F508del incidence has an important impact on the number of patients who are candidates for new therapies. The F508del frequency in this study was a bit higher than the one described in the GBEFC and it was similar to the incidence reported in Southern Europe.

The R1162X is characterized by being most frequently found in northeastern Italy. This may have influenced the incidence in southern Brazil, once the main area of Italian colonization in Brazil was the southern region, and the immigrants were predominantly from Veneto, Northern Italy. Whereas G542X is more common in Mediterranean countries and most parts of Europe, N1303K is found in all Mediterranean regions and it reaches its highest frequency in Tunisia (Mateu et al., 2002). The great number of European descendants in the South of Brazil can explain the highest frequency of these causing variants in the samples of this study (Raskin et al., 2008).

Class I and II causing variants, related to the highest severity profile, were identified in 135 of 184 alleles (73.3 %). The main causing variants found in this study’s sample (F508del, R1162X, G542X e N1303K) are in classes I or II. 

The main limitation of this study is the sample size, as it is a single-center study. However, as we have identified both causing variants in the majority of patients, it would be interesting to describe frequencies and differences among Brazilian regions. Moreover, the frequency of treatable causing variants is a topic of high interest in this new era of personalized medicine.

The main obstacle to the use of *CFTR-modulating* drugs is the high cost of treatment. Due to the countless difficulties, even for creating and maintaining multidisciplinary follow-up centers for CF patients, this discussion becomes more pertinent.

The advent of specific-mutation drugs is of extreme importance, mainly for the most severe causing variant, which is characterized by the very little or no production of the CFTR protein. Increasingly, the focus of the treatment is concentrated on the correction of the genetic alteration that causes the disease. 

In this study, a great number of patients (69 %) are candidates for the use of *CFTR* modulator therapy. Previous studies with the same drugs have presented an increase in pulmonary function, reduction of sweat chloride, a decrease of pulmonary exacerbations, and improvement of life quality. The personalized treatment for CF patients is viable through the recent advances in the development of these drugs. Therefore, the identification of the most frequent causing variants is capable of helping the treatment, boosting the development of target drugs, which are more and more effective.
